# Selections in Children's Diseases; or, a Collection of Select Treatises on Infantile Diseases, for the Use of Practical Physicians

**Published:** 1836-10

**Authors:** 


### Art. III.

*Analekten ueber Kinderkrankheiten: oder Sammlung ausserwählter abhandlungen ueber die krankheiten des kindlichen alters, zusammengestellt zum gebrauche für praktische Aerzte.* Heft I.—VIII. *Stuttgart*, 1834-1836. 8vo.

*Selections in Children's Diseases; or, a Collection of Select Treatises on Infantile Diseases, for the use of practical Physicians.* Parts I.—VIII. *Stuttgard*, 1834-1836.

This is a very valuable and interesting publication, on a plan which we should be glad to see followed in this country, on the present or on other classes of diseases. It consists of numerous monographs which have originally appeared at different times, but all of comparatively recent date, in distinguished practical works, in transactions of societies, journals, &c., in the German and other languages, and most of them by authors of distinction. The foreign articles are translated, and all appear in small neat parts, or volumes of about 150 or 160 pages each, at irregular but short intervals. The first part came out in October 1834 and the eighth is now before us. The articles are, on the whole, remarkably well selected, and bear the names of the most eminent practical writers on the diseases of children in all the countries of Europe, more particularly, however, of Germany and France.

It being our object, on the present occasion, rather to call the attention of our readers to the work than to attempt any analysis of its contents, we shall merely notice a few of the papers contained in it: as, however, we may return to the subject at some future time, we shall restrict our observations to the earlier numbers.

In the 1st vol., page 51, is a well-arranged paper by Dr. Vogel, of Rostock, on the "general Diagnosis of Infantile Diseases," which the author commences by combating the opinion of the obscurity of this class of disorders, and the great difficulty of detecting them. It is undoubtedly true that our knowledge is less advanced on this subject than in many others, yet it is also true that a medical man, with the requisite tact, may arrive at results nearly as accurate, though at the expense of more labour. But, to do this, he must be aware of the value of each sign and symptom,—and their import in children differs from the same in adults,—also quick to detect any variation from the normal condition of any organ. Besides, many of the most important and certain *indicia* in grown persons are either more difficult to obtain in children, or of more questionable validity when obtained. We must therefore seek for more sure guides and other sources of information, and these to a great extent will be found in the general conformation of the body, the equitable adjustment of the different systems, or the predominance of one or more over the rest,—the various temperaments,—the character of the circulation generally,—the disposition to spasmodic action,—the general and local irritability,—the condition of the vital parts, &c. These, with other circumstances of a more local character, are to be taken into consideration, in order to form an accurate diagnosis of any particular disease. Dr. Vogel has taken some trouble in pointing out the anatomical characteristics of different parts of the body in children where they differ from those of the adult; and as this is a very important item in the knowledge requisite, we shall quote his own words.

"1. The distance from the sternum to the pelvis amounts, in the newly-born, to nearly a third part of the whole length of the body; in the adult, only to about one-fifth. At the period when this distance is the greatest in the child, the middle part of the belly is more developed than at a more advanced age. It is also deeper antero-posteriorly than subsequently, when the spine becomes more evidently curved. It is broader, too, because the ribs project more laterally.

"2. The pelvis is much smaller in children, and the os sacrum is curved, with its under part approaching to the os pelvis, the body of which is very short and flattened. The tubera ischii are scarcely as yet developed.

"3. Hence, almost all the abdominal viscera are contained in the middle of the belly, until the pelvis and the space under the short ribs are increased.

"4. The stomach lies nearly perpendicularly, and reaches to the navel; its great curvature is oblique towards the left side, and its smaller curvature towards the right side.

"5. In consequence of this position, the omentum is situated more towards the left side; and it might happen to be mistaken for an obstruction and induration of the colon.

"6. The liver (of a very large size) lies nearly in the middle of the belly, with its anterior border stretching nearer to the abdominal parietes. The left lobe is much larger than in the adult. It is about the fifteenth year that the liver ascends, and then, when the body is recumbent, it is nearly hidden by the ribs. Lastly, the stomach assumes its proper position, and all the other parts their destined relations.

"7. The duodenum lies almost entirely behind the stomach, and its curvatures are very distinct.

"8. The entire mass of the intestines is situated much higher, and pushed more forwards, than in the adult. Should mesenteric scrofula or physconia occur, the child seems to consist almost wholly of belly, and the heart is so pushed up that one can hardly see it.

"9. The spleen is placed more towards the middle of the abdomen, and can be distinctly felt under the short ribs; whereas, in the adult, it lies on the left side in the left hypochondrium.

"10. The urinary bladder is situated almost entirely above and out of the pelvis, and can be plainly discerned when distended with urine.

"11. The uterus and ovaries are above the pubis. The left ovary is ordinarily higher than the right.

"By degrees these circumstances change, and the different organs assume their ordinary positions; but it is easy to see the importance of knowing these peculiarities in our diagnosis of local disease." (Vol. i. p. 53.)

Much more than merely anatomical knowledge is, however, necessary to apprehend these diseases. It is absolutely requisite that the physiology of each organ be understood, in order to detect the commencement of morbid action. The functions of the brain and nervous system,—the vascular system, both of white and red blood, the lungs, the abdominal viscera, the skin, the cellular tissue, &c., must be carefully studied; and, when once our information on these points is extensive and accurate, we shall have but little difficulty in appreciating deviations from health, even in children, where we cannot avail ourselves of their intelligence. All this is dwelt upon in detail by M. Vogel, and we must refer our readers to his paper: but there is one point we cannot pass over so slightly. In the phænomena of functional disturbance, to which we have just referred, a few points excepted, there is no very wide difference between similar processes in children and in adults, but in one particular, and a most important one, where morbid action is in question, childhood wholly differs from more advanced age, viz. that it is a period of development. Not only have the various organs their ordinary functions to fulfil, but they have also to provide for the collective development of the body. The consequence is, that we find an increased activity of certain organs and an exaltation of vitality generally. The irritability and sensibility are so exquisitely marked at this period, and the general and local excitement so intense, that but a very delicate line is the limit of health and disease; and we cannot be surprised either at the frequency of certain affections, or at the slight causes which suffice to produce them.

"The most important epochs of infantile life must be considered, in order to enable us to comprehend the entire subject, so as to obtain a successful general diagnosis of the diseases of children. These are *the periods of development*, during which the vitality in certain hitherto quiescent and powerless organs is exalted by the increased physical and dynamical growth and development, and which has a most powerful influence upon all the functions of the body and upon their mutual relations.

"One of these important periods, which is fatal to many children, is the time of dentition. Since it ordinarily preserves its regular stages, we may conclude, from calculation, that many accidents are attributable to this cause, even when it does not announce itself by local symptoms.

"In all processes concerned in the evolution of the body, the vegetative power or vital action of the organs concerned is necessarily exalted. This shews itself by increased vascular action in the part, afflux of the fluids," &c. (P. 62.)

After noticing the sympathies of distant organs with morbid action, and pointing out the necessity for their careful investigation, our author proceeds to consider the causes and course of the more ordinary diseases, especially with reference to food, medicine, air, clothing, exercise, &c. Considerable stress is laid upon the cry of children, as assisting our diagnosis, and Billard's distinction is adopted. We may observe the cry which takes place during expiration, which is more or less clear, sounding and continuous, and the cry during inspiration, a kind of echo of the former. The characters of these two cries are very different, and have different significations. The younger the child, the shorter the echo, which becomes more audible with advancing years, and the intensity of which appears in inverse proportion with that of the cry of expiration. If the suffering be acute, and the child fresh and strong, the cry of expiration predominates; but, if exhausted or fatigued, the cry of inspiration, as in sighing or sobbing. It is probable that a reference to Dr. Calvert Holland's views of the relations of expiration and inspiration would throw light upon this subject. In very young children, crying is generally (but not always) accompanied with tears. For some ingenious views on this subject we must refer to the work of Billard before noticed.

The remainder of the essay is occupied with considering the physiognomy of children as a ground of diagnosis, and as we proceed next to notice Dr. Ph. A. Pieper of Paderborn's paper on this subject, we may as well incorporate the two. During infancy, the countenance is far more the index of physical than of mental operations. Owing to the quiescence of the intellectual faculties, it is more influenced by the bodily condition. For some years, in fact, it is indicative of sensation rather than thought, and these sensations are mainly drawn from physical sources. Here, then, is a wide and certain field of knowledge laid open to our research; but this is not all—as in after-life, certain regular physiognomical combinations are involuntarily employed to express internal operations, so, in early life, analogous expressions of countenance point out certain sensations merely physical. Jadelot was perhaps the first to investigate this subject systematically, and he has been followed by Eusebe de Salle, Otto, Froriep, Meissner, Billard, &c. As the experience of the present writers confirms Jadelot's views, and as they adopt his system and nomenclature, at least to a certain extent, and moreover as we have reason to believe that this source of diagnosis is less attended to than it deserves in this country, we shall here give a brief abstract of what they advance on the subject, although it is now many years since it was first submitted to the profession by its original proposer. We think it right, at the same time, to guard the young practitioner against relying too strongly on this method of diagnosis, which, after all, although founded on physical grounds, is merely symptomatical in its nature, and of quite different import from the signs of diseases strictly termed *physical.*

After extensive observation and analysis, Jadelot fixed upon certain traits or features of which the development accompanied certain states of disease, and from the regularity of their occurrence, he regarded them as pretty sure grounds of diagnosis. MM. Vogel and Pieper describe, 1, the "trait occulo-zygomatique," extending from the outer canthus of the eye to the zygomatic arch; 2, the "trait nasal" and the "trait genal," which together form a line from the side of the nose downwards under the eye to the edge of the malar bone, including a slight portion of the outer line of the orbicularis oris in its semicircle; 3, the "trait labial." It is evident that this map of the face embraces the elements of many of the near determinate expressions, and many of those which predominate in disease. The precise affections which these traits are supposed to indicate are thus laid down by M. Pieper, (p. 8, 3d part.) "The first 'trait' is observed to be strongly marked in all diseases which have their seat primarily in the brain and nerves. When it alone is prominent, these parts only are diseased. But it is frequently connected with the second and third 'traits,' and this complication points out some inflammation either of the chest or belly. When the first is superadded to the two latter, a primitive lesion of the chest or abdomen is indicated which has taken on a nervous character; and in these cases convulsions often follow. The early or late appearance, then, of this first trait indicates a primary or secondary brain affection.

"The 'trait nasal' refers always to disease of the abdomen: this must be carefully distinguished from a similar line observed in healthy children, whose cheeks are fat. The circumstance, however, that the physician has to do with a sick child in whom the thinness of the face gives rise to more pronounced features, together with the other symptoms, will prevent this error. This 'trait' is well developed in dysentery. The 'trait genal' is more evident when the stomach and intestines are affected at the same time; the chin is protruded, the lips are drawn tight across the teeth, and the mouth is made wider; this constitutes the 'face grippée,' which, often as we have heard of it, has never before been analysed. Should then diseases take on a nervous character, the first 'trait' will be superadded. For this reason the appearance of the 'trait genal' in conjunction with the 'trait occulo-zygomatique,' together with paleness of the face and redness of the eyes, are signs of worms; since the intestines are primarily and the brain secondarily stimulated by them. The 'trait labial' is most evident in diseases of the chest."

But, whilst admitting the truth of these observations, M. Pieper is aware of their limited extent; and, in order to supply this deficiency, he proposes to consider what he calls the "quantitive relation" of the physiognomy. The parts constituting this relation are divided into hard and soft: "the hard include the bones of the head, more or less; and the softer are given in the other parts of the countenance, and exhibit in the muscles the freest and most powerful organs for the formation of the physiognomy." In pursuance of this view, he proceeds to examine the cranium in its relations to the countenance; then the bones of the head in conjunction with the face; and, lastly, the bones of the face by themselves. The importance of such considerations is apparent when we recollect that the size and shape of the skull are a very accurate representation of the size and shape of the brain; that it is an extremely active and extensive organ in children, very liable to be sympathetically affected, and indicating, by its size or relative proportions, many of the diseases to which it is obnoxious. Many cases of convulsions and hydrocephalus, or even slighter instances of the predominance of the nervous temperament, illustrate and establish this point.

The softer parts consist of certain features subject to both external and internal influences: the forehead may be smooth and fair, or corrugated; the depth of the orbit varies from different causes; the eyes, the nose, and the mouth, are capable of infinitely varied expression; and, if we add to the information obtained from hence, that derived from the consideration of the 'traits' already described, and which should be observed as well when the child is asleep as awake, we shall be in possession of knowledge which will assist greatly in enabling us to make an accurate diagnosis.

In the first part, there is a paper by Dr. Tortual, of Munster, on the treatment of diseases of children, which contains some useful observations along with much more matter which is very common-place and uninteresting. The object of his paper is to moderate the quantity of medicine given to children, not merely because so much is needless, but because, if it act at all, it will probably interfere with the regular course of the disease, and may thus deceive the medical attendant by masking the diagnostic symptoms. There are two periods when great discrimination in the use of remedies (at least the more active ones,) is necessary, viz. in the very outset of the disease, before it has assumed a definite form, and after the violence has somewhat subsided, when the patient is verging toward convalescence. In the first case, we may disarrange the true development of the disease, and deprive ourselves of the true indications of cure; and, in the latter, we may render recovery more tedious and less complete. This applies peculiarly to children, in whom we see a greater effort made by the constitution to throw off disease, and in whom its course is even more defined than in adults. The arguments of M. Tortual are derived from the difficulty of changing the regular progress of disease; from the efforts of nature to disembarrass herself of noxious matters; and from the ill effects of stimulating medicines or food during convalescence. Further illustrations are drawn from that class of secondary affections which depend upon some more occult primary process, such as the sympathetic irritation of distant organs during dentition, and which cannot be interfered with but at the risk of greater damage to the constitution.

We agree with the author of this paper in deprecating the excessive use of medicine in these cases, and we doubt not that much subsequent delicacy of constitution may be traced back to this cause; but we cannot but think, at the same time, that he is disposed to run into the opposite extreme. It requires the nicest discrimination to know when and how long to withhold our remedies, and nothing can give this tact but a prolonged and very accurate observation of the course of disease, its different phrases, the succession of its stages, &c. Without this our "médicine expectante" will be as mischievous as the opposite extreme, and we shall see our patient snatched away when a little more intelligence and promptitude might have cut short the disease.

The next paper which we shall notice is a memoir by Prof. Mende, of Göttingen, in "the Asphyxia or apparent death of new-born Infants." He first describes those cases where life seems to stand still, if it be not utterly extinguished, where no external motion distinguishes the "viable" infant from a dead one; and, after tracing this state primarily to the condition of the brain and nervous system, he observes that, as it originates with these organs, so it is by acting upon these, either directly or indirectly, that we may hope for restoration. P. 91, Part I., he describes the influence which can thus be exercised as threefold: "1. Through impressions which apply immediately and operate definedly, on account of a special relation to the nervous system. 2. Through consensual influence originating in parts with which it (the nervous system) has a nearer or more distant reciprocity; as, for instance, the lungs, upon which it is as dependent as any other part for the conversion of its venous blood into arterial. 3. Through the reaction of the whole system upon the nervous system as a part of that whole."

For the better understanding of the subject the Professor divides these asphyxiated or apparently dead children into three classes.

"The first, including every species which arises immediately from the state of the whole nervous system of the fœtus, whose external sensibility is not as yet fully awakened, and in this respect occupying a lower grade than usual. 2. The second class are those depending merely on the brain, and which, according to what has been already observed, are caused either by a gradual and continued or a sudden and more severe pressure on its periphery. 3. The third includes those cases which depend upon the inadequacy of certain organs, without whose activity the influence of the nervous system in new-born infants cannot be exerted upon the rest of the body. These organs are the lungs and the vascular circle." (P. 94.)

The causes of the asphyxia may be easily arranged according to this division. Amongst those of the first class, we find weakness in either parent, sickness or bad nourishment of the mother during gestation, deficiency of the placenta or navel string, diseases of the fœtus, sudden detachment of the afterbirth with hemorrhage, premature delivery, fainting, asphyxia, or death of the mother. The causes of the second may be divided into those which occur *before* delivery; such as, long continued pressure upon the head or a sudden compression; and those which happen *during* the birth; such as pressure on the head by the pelvis merely, from some other part descending along with the head, from the use of instruments, or from congestion arising from the twisting of the navel string around the neck. With regard to the latter, it is enumerated as a cause of asphyxia by all writers as well as our author, and we think without sufficient examination: of course the way in which such an effect is supposed to be produced, is by the shortening of the cord, and the consequent tightening which the expulsion of the child occasions. Now, a little careful observation would shew that this is a very rare case. Out of about fifty cases we have found that the ordinary length of the cord is one foot five inches, or one foot six inches; but in the same number of cases we have seen but a single example of a cord of that length being twisted round the neck: those which were so twisted were generally upwards of one foot eight inches, and the number of coils was *always* in proportion to the length of the funis. But this increase in length readily admits of the expulsion of the child without more stress on the cord than in those cases where it is not coiled round the neck, and consequently without more risk of asphyxia.

With regard to the injury inflicted by obstetrical operations, Prof. Mende mentions the fact, that a child may suffer a considerable loss of cerebral substance, and yet be born alive; whereas continued pressure on the surface of the brain causes coma, asphyxia, or death. Dr. Beatty, of Dublin, and others, have related cases where the crotchet and perforator had been used, and yet the child cried loudly after delivery; and we saw a case recently where the child was delivered by those instruments, and yet it lived three weeks after delivery, troubled however, as the mother observed, with nine-day fits.

To this class also belong those cases of apoplexy which we so often meet, whether there be effusion of blood on the brain, or merely arrest of the circulation; but, though considerable details are given, we do not see noticed a circumstance which is a very frequent cause of this accident.* It not unfrequently happens in labours that are somewhat protracted (first labours especially,) that the uterus, fatigued by its extraordinary efforts to expel the head, ceases to act for some time after the completion of so much of the delivery. In this quiescent state it may remain for half an hour or more; but a far less space of time suffices to destroy the child by apoplexy. If only a few minutes have elapsed, although the face may be livid and distorted, it is possible in some cases to save the child by allowing some blood to flow from the funis; but, after a longer period, when apoplexy seems to have taken place, there is scarcely a chance of recovery. However, nothing is more simple and easy than the prevention of such consequences. Slight traction should be made by the accoucheur in order to excite the uterus to expel the child; and, if this be insufficient, the child must be extracted. There is no difficulty nor any danger of laceration in doing this, as the widest part of the child has previously passed the external orifice, and the only

* We are happy to add the testimony of so experienced a practitioner as Dr. Collins, of Dublin, on this important but neglected point. In his Practical Treatise on Midwifery, noticed in our last number, he observes (p. 5,) "Although, generally speaking, no assistance whatever should be given, yet I have known several instances where the child was lost by adhering too strictly to this rule, where uterine action was tardy in returning after having expelled the head. When therefore, in three or four minutes after the head has been protruded, the pains do not return, particularly if the face be observed to be very livid, or still more when the child makes efforts to breathe, we should give gentle assistance so as merely to excite uterine action."—Rev.

objection that can reasonably be urged against this plan is the risk of uterine hemorrhage; against which we can guard with tolerable certainty by making the nurse follow down, and press firmly upon the receding uterus, and by using a tight binder with compresses when delivery is completed.

The symptoms observed in this infantile apoplexy are well known, and may be found in all standard works on midwifery.

The third class includes every species of suffocative asphyxia arising from delayed or obstructed respiration on account either of physical debility, the non-penetration of air into the lungs, a deficient supply of it, or its bad quality. To this class are referred all malformations or intra-uterine diseases of the lungs or wind-pipe, and obstructions from foreign matters in any portion of the air tubes.

With regard to the treatment of these three classes, nothing new is suggested. In all cases of determination to the head, the loss of blood is our most powerful remedial agent. The quantity to be lost must be determined by its effects upon the constitution and state of the child. Where the head is not primarily affected, the removal of any impediment to respiration, and affording plenty of pure air,—the artificial inflation of the lungs,—the exhibition of stimuli,—irritation of the surface by friction or slapping the back, hands, or feet,—cold effusion,—the warm bath, &c. are the principal means to which we should have recourse.

In Vol. II. is a very excellent paper by Dr. Heyfelder, of Sigmaringen, extracted from his "Observations on Diseases of New-born Children," on the induration of the cellular tissue. It appears to be the result of his investigations on the subject in the French hospitals, where the disease is much more frequent than in this country. In 1822, there were three hundred cases in the "Hospice des enfans trouvés;" and, in 1823, there were four hundred. In Great Britain it is comparatively a rare disease, and in Germany almost unknown. The disease arises in new-born children generally during the first five days, and frequently much earlier, from eight to twelve hours after birth. It is not confined to newly-born infants, however; for Naudeau observed it in children four months old, and Henke saw similar symptoms in a girl of twenty years. It severally commences in the lower extremities, which lose their natural colour, and become yellowish or reddish yellow, and subsequently deep red or a reddish blue. The limbs soon become tense and cold. These symptoms extend themselves to the thighs, the parts of generation, and the belly as high as the navel string; the upper extremities, and the neck and face, are then affected. The hardness of the surface increases in proportion to the diminution of the heat of the parts, until the skin entirely ceases to pit upon pressure, and the limbs feel stone cold, like those of a dead body. The motions of the parts affected is greatly impeded; the head is turned to the right or left with difficulty and apparent pain, and the child appears deaf. The eyes are closed, and the upper eyelid much swollen; the face is full, the cheeks are hard and shining. Towards the end of the disease, the mouth is spasmodically closed, the lips are tense and dark coloured, and the angles of the mouth yellow. The child can scarcely open the mouth, and is unable to suck. The respiration is difficult, the lungs appear to remain inactive in the chest; the cry is feeble and piping.

These symptoms are somewhat mitigated by the application of warmth; but, the moment the child is removed from the warm bath, they all return as bad as before. If the air to which the child is exposed be cold and moist, the symptoms proceed more rapidly, and death follows in a shorter time. At the commencement of the disease, the pulse is slow and feeble, but perceptible; at a more advanced stage, it ceases altogether in the extremities, but may be felt the longest in the axillary arteries. The action of the heart, which is evident in the beginning, is not perceptible towards the end, even with the stethoscope.

The termination is seldom favorable; but, in the few cases which recover, we observe the disappearance of the peculiar colour and the hardness; the respiration is free, although still performed imperfectly; the limbs are more moveable, the action of the heart and arteries more perceptible; the bowels are less free, and the eyes are unclosed. The convalescence is very tedious, and the patient is extremely liable to relapses.

In the fatal cases, in which death generally takes place before the seventh day, all the symptoms increase, especially the coldness, hardness, and emaciation; swallowing is impossible; the voice ceases; regurgitation of the fluid in the stomach (sometimes mixed with blood,) takes place, and sugillation of the back and upper parts of the thigh.

With regard to the appearances after death, if we make an incision into the swollen and hard parts, we find in the cellular tissue a serous glutinous fluid, sometimes yellowish, at others grey and dark-looking. According to Auvity, it coagulates in boiling water, and remains fluid in cold. The fluid often differs in different parts of the same subject; and in one case Paletta found effusion of blood instead of this serous fluid.

The fat is yellow, orange, or dark-coloured. The lymphatics are distended, especially those in the mesentery. The veins are obstructed by a black half-coagulated blood, particularly the superficial ones, and those of the spinal marrow.

Here and there are spots of a yellow colour in the brain, and a yellow fluid is also found, which is less viscid than that contained in the cellular tissue.

The pharynx and epiglottis are distended by this fluid, and the lungs by black blood; they are hard, dark-coloured, and as it were marbled and hepatized. Occasionally but one lung is found in this state when the infant has lain only on one side. The yellow effusion is also found in the pericardium and cavity of the thorax and abdomen. The liver is distended in like manner, but the secretion of bile is not suspended. Occasional red spots, resembling inflammation, are observed on the mucous membrane of the stomach and intestines; and a curious remark has been made on the comparative length of the intestinal canal, and especially of the small intestines. The remark is due to M. Leger, *elève interne* of the Foundling Hospital at Paris.

Dr. Heyfelder observes,

"The length of the healthy intestinal canal in new-born children is about ten feet at the utmost; in those who died of inflammation of the belly, it amounts to fourteen or fifteen feet. The following table will shew its length in those who die of induration of the cellular tissue.

"In one child it was found to be 4 feet 6 inches.

| | | |
|---|---|---|
| 2 children | — | 4 — 10 — |
| 5 — | — | 5 to $5\frac{1}{2}$ feet. |
| 7 — | — | $5\frac{1}{2}$ to 6 |
| 9 — | — | 6 to $6\frac{1}{2}$ |
| 10 — | — | $6\frac{1}{2}$ to 7 |
| 15 — | — | 7 to $7\frac{1}{2}$ |
| 16 — | — | $7\frac{1}{2}$ to 8 |
| 14 — | — | 8 to $8\frac{1}{2}$ |
| 9 — | — | $8\frac{1}{2}$ to 9 |
| 5 — | — | 9 to $9\frac{1}{3}$ |
| 3 — | — | $9\frac{1}{3}$ to $9\frac{3}{4}$ |
| 2 — | — | $9\frac{3}{4}$ to 10 |
| 1 — | — | 10 |
| 1 — | — | 11." (P. 50.) |

The author does not attempt to explain this remarkable deficiency, nor does he state any thing which would lead us to attribute it to the disease itself.

The results of chemical analysis of the fluid effused in the cellular tissue by M. Chevreul are given.

"The yellow serous fluid coagulated as soon as heat was applied, like the serum of the blood, and was slightly alkaline mixed with alcohol; an albuminous and somewhat yellowish precipitate was thrown down, which was soluble in alcohol. When the fluid was filtered and evaporated, there were found salts, a reddish principle like drops of oil, and another matter resembling in colour the resin of the bile.

"The bile in the gall-bladder contained gall, resin, and a remarkable quantity of picromel.

"From the heart M. Chevreul obtained cruor, fibrine, and albuminous matter. The serum yielded, with alcohol, the same reddish, yellow, and green matter as the fluid in the cellular membrane.

"A special peculiarity of the serum of the blood and the yellow serous fluid, is that when mixed in a vessel they change in a short time into a glutinous mass, which, upon pressure, separates into a membrane and a yellow fluid. From these results M. Chevreul concludes,

"1. That the yellow fluid found in the cellular tissue is formed in the blood, simply separated from it, and effused into the cellular membrane, as the colourless serum of the blood is in dropsy.

"2. The coagulability of this yellow fluid explains the hardness of the distended cellular tissue, and why it does not escape when an incision is made into the skin.

"He adds his belief that the yellow colour depends upon the bile, since all its animal matters are found in the blood.

"If we compare the appearances of this disease with the information obtained by *post-mortem examination*, they would seem to be the product of an incomplete circulation of blood in the obstructed lungs, and consequently of a deficient oxygenation thereof, which is necessarily followed by a decrease of heat."

The latter is the view of the essential nature of the disease adopted by the author: the diminution of the rapidity and perfection of respiration is followed by a proportionate diminution in the rapidity of the circulation, but the development of animal heat depends on the deoxydation of the blood in the capillaries; if, therefore, the circulation in this system be less rapid, the evolution of heat will be less. The quality of the blood in circulation will be further deteriorated, because of the open state of the foramen ovale allowing the transit of the venous blood through it, when the passage through the lungs is obstructed.

The pulmonary obstruction also gives rise to the congested state of the abdominal organs. The effusion into the cellular membrane takes place in consequence of the arrest of the capillary circulation.

The causes of the disease may be stated generally to be those which interrupt the functions of the skin; especially cold and moist weather, or damp localities. Souville observed it in the neighbourhood of Calais, which is a part of the year under water. Auvity says that it arises in the cold and damp weather in the beginning of autumn, prevails extensively during the winter, and disappears when the warm spring weather commences.

Any thing which interrupts the circulation and respiration may give rise to it; thus, extreme cold, though without wet, has been found favorable to its production.

These causes, however, do not explain entirely its great frequency in Foundling Hospitals, (that of Paris especially;) and there can be no doubt that the deprivation of their natural nourishment is one of the most important. We know that the mortality amongst spoon-fed children is much greater than amongst those brought up at the breast; and, if we couple with this the fact that this disease attacks the children in these hospitals generally at a period when they are quite unaccustomed to artificial nourishment, and less able to bear it than subsequently, we shall see good reason to place it amongst the most influential causes of this disease.

With regard to the treatment, the author gives us the opinions of different authorities rather than the results of his own experience. As a preventive, Auvity advises that sudden changes of temperature be avoided; that children should be washed in warm water, immediately dried, and rubbed with flannel; and that all unctuous and spirituous applications be avoided.

Gölis attacks the disease with mercury successfully, but Doublet has seen it fail. Souville recommends warm baths and fomentations; Andry repudiates them. Moscati and others, who regard the disease as a spasm of the cellular tissue, estimate very highly the use of camphor, musk, &c. In fact, the opinions of these authors rest more upon theoretical views than upon their experience. Warm baths, frictions, blisters, with small doses of mercury, appear to have succeeded to a certain extent in the hands of Auvity, Breschet, and Paletta: the latter found benefit in some cases from the loss of a small quantity of blood. Of course, we should always, where this is practicable, have recourse to breast-milk as a substitute for artificial nourishment.

The next paper we shall notice is one by C. W. Hufeland "on the Intra-Uterine Diseases of the Fœtus." We cannot feel much surprise that a being whose vascular system is in a state of activity, and whose nervous system is only in part quiescent, whose organs are exercising their respective functions or acquiring their due development, should be subject to those variations from the normal condition which are observed, and still less when we reflect upon the mechanical and vital relation which the fœtus in utero bears to the mother, although the intimate operation of these latter causes may be hidden from us. The investigation of the subject has been undertaken by our author with considerable sagacity. He first considers the various modes by which the fœtus may be influenced, not merely as pathological conditions, but also remedially:

1. By means of the materno-fœtal circulation; for, although we do not believe in the direct transmission of the maternal blood, there can be no doubt that a change in the quality of this fluid has a proportionate influence in the production of fœtal disease. We know that, amongst other causes of these changes, the diet of the mother holds the first place; and hence we acquire one means of influencing the state of the fœtus.

2. The nervous system of the mother is also capable of communicating impressions to the child; as, for instance, sudden mental shocks, fright, &c. have been known to destroy the fœtus.

3. Mechanical means, such as blows on the abdomen of the mother, falls, shocks, &c. may injure or destroy the fœtus.

4. The common natural agents, such as warmth, electricity, magnetism, &c. applied to the fœtus through the body of the mother, may produce these effects upon it.

5. Communication of disease from the mother to her infant may take place, as is observed in children born with syphilitic affections, &c., and the type of any organ or system of the mother may be transmitted to the fœtus. So much for the way in which mischief may be done to the embryo; the question naturally arises, whether we cannot influence the child medically by some of these modes; and our author concludes (p. 87, vol. ii.) that these are the following means in our power by which we may act upon the child in the womb:

"1. By the increase or diminution of (maternal) nourishment. 2. By the increase or diminution of the rapidity of the circulation. 3. By changing the quality of the nutrition and air. 4. By mechanical means, as altered position of the mother, bandages, &c. 5. By the natural agents. 6. By medicines, and these of three kinds, either those which act upon the nervous system, upon the circulation, or by simple penetration, as the most fluid, such as æther, ammonia, musk, camphor, &c. appear to do. 7. By mental impressions."

The second part of this essay is devoted to the investigation of the peculiar diseases to which the fœtus has been found liable: the first class includes malformations and monstrosities; the second, atrophies; the third, hypertrophies; and the fourth, dyscrasies, diseases or dispositions to disease, such as scrofula, syphilis, (of which some striking examples are given,) the exanthemata, &c.; the 5th, nervous and mental disorders; the 6th, accumulations of fluid; the 7th, vascular congestions and inflammations; the 8th, skin diseases; the 9th, worms; the 10th, disorganizations, pseudo-organizations; the 11th, mechanical injuries; the 12th, death previous to birth. The principal causes of the latter are stated to be fright, strong mental emotion, accidents or diseases on the part of the mother, deficient vital energy on the part of the child, and congestion of the uterine system. All these circumstances are tolerably well known, and examples may readily be met with; therefore we shall not dwell upon them, merely observing in passing, with regard to the death and expulsion of the ovum at an early period, that, in seven cases out of ten, the cause is local, and to be found either in disease or malformation of the ovum or fœtus; and consequently abortion, instead of an accident to be avoided or postponed by any means in our power, is in fact the best thing that could happen under the circumstances, as it frees the uterus from that which might form a nucleus for disease.

The third and last portion of this paper is occupied with the practical application of the means described in the first part, to the preservation of the life and health of the fœtus in utero. These are, 1. By attention to regimen. Inasmuch as congestion is one of the most frequent causes of injury, we must counteract it by the abstraction of blood, by rest, and the horizontal posture. The author states that he has often found the movements of the child increase in strength after bleeding the mother. Again, the child is often destroyed by the feebleness of its constitution; and we are advised to remedy this by stimulating frictions to the abdomen of the mother, or we may thus apply laudanum if the irritability or disposition to convulsive action be excessive. The administration of ice or of medicated waters (those of Pyrmont and Driburg are named,) is spoken of as extremely useful.

2. By suitable and normal development and nourishment. By increasing or diminishing the nutrition of the mother, M. Hufeland conceives that we can regulate the degree of fœtal growth.

3. By strengthening the constitution of the fœtus, and removing any nervous weakness; and this is to be done mainly by the mother using simple nutrition, avoiding stimulants, and regulating her diet exactly according to any idiosyncrasies of her own constitution.

4. By purity of the fluids, and removing any disease under which the mother may labour.

5. By accustoming the mother to impressions of beauty. The ancients were in the habit of placing before the eyes of pregnant women the most exquisite paintings; and M. Hufeland remarks that, in Catholic churches, where adoration of the Virgin is performed with peculiar earnestness by pregnant women, there is something *Madonna*-like about the countenances of the female offspring,—an observation in which perhaps fancy has some share.

We have preferred giving a condensation of M. Hufeland's Essay, on account of its importance, rather than entering into detail upon any particular part. Those anxious for more minute information we refer to the original itself, promising them that they will be amply repaid for their trouble.

In the Third Volume is a valuable paper on Aphthæ and Muguet, by Dr. Naumann, of Bonn, which, however, we can do little more than point out to our readers. The two disorders, so different and yet so liable to be confounded, are well described, and the distinction between them is accurately drawn; the one being a secretion upon the mucous membrane of the mouth and tongue, and the other an elevation of that membrane in the form of small blisters, which, if broken, reveal small superficial ulcers. The state of the constitution has probably much to do with the production of each variety, but especially in aphthæ; and, we consequently, find local remedies more universally successful in muguet. These, however, should not be alone depended upon; but we may at the same time exhibit a combination of alterative medicines with purgatives. Emetics are also recommended in the commencement. Probably the best local remedies are borax and honey, or a mucilaginous gargle with alum or dilute sulphuric acid.

We cannot conclude our review without repeating our wish that we had a similar collection of monographs on diseases of children by British authors. There are numerous and very valuable essays scattered through the various periodical works or published separately; but we have no standard book on diseases of children.

